# Robust Material Properties in Epitaxial In_2_Te_3_ Thin Films across Varying Thicknesses

**DOI:** 10.1002/smll.202508738

**Published:** 2025-11-14

**Authors:** Maximilian Buchta, Felix Hoff, Lucas Bothe, Niklas Penner, Christoph Ringkamp, Thomas Schmidt, Jan Köttgen, Timo Veslin, Ka Lei Mak, Jonathan Frank, Dasol Kim, Matthias Wuttig

**Affiliations:** ^1^ Peter‐Grünberg‐Institute – JARA‐Institute Energy Efficient Information Technology (PGI‐10) Wilhelm‐Johnen‐Straße 52428 Jülich Germany; ^2^ Institute of Physics IA RWTH Aachen University Sommerfeldstraße 52074 Aachen Germany

**Keywords:** chalcogenides, coherent phonons, metavalent bonding, molecular beam epitaxy, optical properties

## Abstract

Sesqui‐chalcogenides serve as a critical bridge between traditional semiconductors and quantum materials, offering significant potential in applications such as thermoelectrics, phase change memory, and topological insulators. While considerable attention has been focused on antimony‐ and bismuth‐based compounds, characterized by substantial property changes upon reduction in film thickness, indium‐containing sesqui‐chalcogenides like In_2_Te_3_ are emerging as promising candidates for photovoltaics and electronic devices. However, the effects of film thickness on the properties of In_2_Te_3_ remain largely unexplored. In this study, we investigate high‐quality In_2_Te_3_ thin films grown by molecular beam epitaxy on Si(111) substrates across a thickness range from 2.7 to 24 nm. X‐ray diffraction, reflective high‐energy electron diffraction, and atomic force microscopy are employed to analyze both the crystal structure and film morphology. Additionally, broadband optical spectroscopy alongside femtosecond pump‐probe measurements and Raman spectroscopy are utilized to assess optical and vibrational properties, respectively. This analysis reveals that material properties exhibit minimal dependence on film thickness ‐ contrasting sharply with behavior observed in other chalcogenides such as Sb_2_Te_3_, Bi_2_Se_3_, or GeTe. This phenomenon can be attributed to the covalent bonding present in In_2_Te_3_, which differs from that in its antimony‐ and bismuth‐containing counterparts.

## Graphical abstract

Epitaxial In_2_Te_3_ thin films grown on Si(111) substrates display remarkably robust structural, optical, and vibrational properties across a broad range of thicknesses. Unlike metavalent chalcogenides (Sb_2_Te_3_ or GeTe), these covalently bonded films maintain their intrinsic characteristics even at the nanometer scale. Comprehensive characterization reveals minimal dependence on thickness, highlighting In_2_Te_3_ as a stable platform for nanoscale devices.

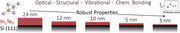

## 1 Introduction

The family of sesqui‐chalcogenides plays a pivotal role in connecting conventional semiconductors and emerging quantum materials, exhibiting applications as thermoelectric materials,^[^ ^1^, ^2^ ^]^ phase change memory materials,^[^ ^3^, ^4^ ^]^ and topological insulators.^[^ ^5^, ^6^ ^]^ While significant attention has been directed toward antimony and bismuth‐based sesqui‐chalcogenides, indium‐containing compounds have recently gathered considerable interest within the field of photovoltaics,^[^ ^7^ ^]^ ferroelectric data storage,^[^ ^8^, ^9^ ^]^ and electronic devices.^[^ ^10^, ^11^ ^]^ Notably, In_2_Te_3_ has emerged as a promising material for broad‐spectrum photodetectors^[^ ^12^, ^13^ ^]^ and photovoltaic power generation.^[^ ^7^, ^14^ ^]^

Despite its technological relevance, research into the fundamental understanding of thin film crystal structures and phase transitions remains in its early stages. The existing studies predominantly focus on bulk crystals^[^ ^15^, ^16^, ^17^ ^]^ even though high‐quality thin films are crucial for modern compact devices. Recently, research has shifted toward growing thin films using various deposition techniques across a range from a few to hundreds of nanometers (nm).^[^ ^11^, ^12^, ^18^ ^]^

As demonstrated for certain chalcogenides, material engineering based on film thickness is particularly effective when the thickness is reduced below 10 nm.^[^ ^19^, ^20^, ^21^, ^22^, ^23^ ^]^ This phenomenon has been linked to a newly proposed type of chemical bonding termed metavalent bonding, which involves the interplay between electron localization and delocalization.^[^ ^19^, ^20^, ^21^, ^22^, ^23^, ^24^ ^]^ Such bonding results in high Born effective charges (Z^*^), elevated dielectric constants, near‐metallic conductivity, large mode‐specific Grüneisen parameters for optical phonons, unusual bond‐breaking behaviors, and effective coordination numbers (ECoN) that deviate from the octet rule.^[^ ^25^, ^26^, ^27^ ^]^ These phenomena arise from the competition between electron localization and delocalization, not found in covalent crystals.^[^ ^28^, ^29^ ^]^

Therefore, this study aims to investigate whether this phenomenon applies to In_2_Te_3_ as well.

To achieve this goal, high‐quality thin films with thicknesses ranging from 2.7 to 24 nm were grown on Si (111) using molecular beam epitaxy (MBE). The structural properties of these samples were analyzed concerning their thickness using low‐energy electron diffraction (LEED), reflection high‐energy electron diffraction (RHEED), X‐ray diffraction (XRD), X‐ray reflectometry (XRR), and atomic force microscopy (AFM). Their thickness‐dependent properties were investigated using broadband optical spectroscopy to obtain the dielectric function. Additionally, femtosecond pump‐probe measurements combined with Raman spectroscopy were employed to characterize the lattice dynamics and light‐matter interactions. Finally, density functional theory (DFT) is used to gain insights into the chemical bonding mechanism within the material. This comprehensive approach ensures a unified interpretation of the experimental findings.

## 2 Epitaxially Grown In_2_Te_3_ Thin Films and Their Atomic Structure

Indium Telluride crystallizes in multiple stable configurations, including, among others, InTe, In_2_Te_3_, In_3_Te_4_, In_2_Te_5_, and In_4_Te_3_.^[^ ^30^ ^]^ Therefore, MBE is employed due to its ability to provide excellent stoichiometric control. The films were grown on the unreconstructed Si (111) 1 × 1 surface under Te‐rich conditions. RHEED was utilized for in situ monitoring of the growth process. Subsequently, the samples were capped in situ with Al_2_O_3_ using sputtering. Further details can be found in the Experimental Section.

**Figure 1:**
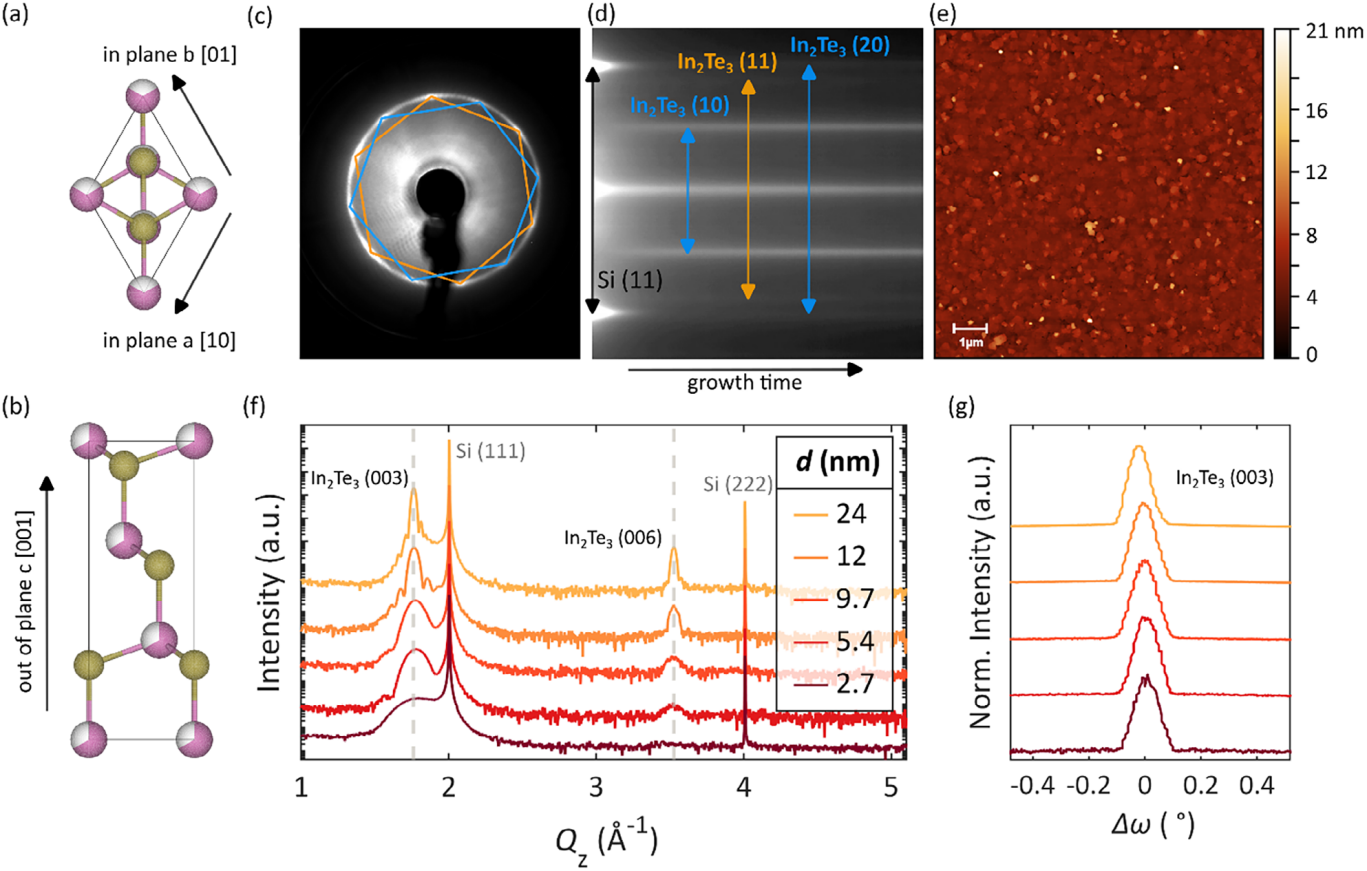
Structural analysis of the In_2_Te_3_ thin film series. The crystal structure of In_2_Te_3_ (111) is illustrated in both directions: a) in‐plane and b) out‐of‐plane, with the In atoms in pink and the Te atoms in gold. Panel c) displays the LEED pattern (*E* = 59 eV) where the two rotational domains are marked in blue and orange; d) shows the corresponding streaks from the RHEED growth video. Film streaks are labeled according to their respective in‐plane diffraction order. The AFM scan in e) confirms a high surface quality with an RMS roughness value of 1.05 nm. The XRD diffractograms and rocking curves of the In_2_Te_3_(003) peak are displayed in f) and g), which confirm a high texture quality alongside phase purity.

In_2_Te_3_ adopts the ZnS crystal structure,^[^ ^31^ ^]^ belonging to space group $F\bar{4}3m$, with a lattice constant of 6.16 Å (see Figure S1, Supporting Information). To compensate for the additional electron contributed by indium compared to zinc, one‐third of the indium sites is vacant. The films are oriented along the (111) orientation of this crystal structure, resulting in an in‐plane lattice constant of *a* = 4.36 Å and an out‐of‐plane lattice constant of *c* = 10.67 Å. The crystal structure of In_2_Te_3_ along the (111) growth direction is illustrated in **Figure** **1**a for the in‐plane direction and Figure 1b for the out‐of‐plane direction.

Figure 1c presents the LEED pattern of In_2_Te_3_ films, revealing two distinct in‐plane rotational domains marked in blue and orange. A ring connecting these diffraction spots indicates an angular misalignment relative to the substrate – a phenomenon known as fiber texture. Yet, two rotational domains are predominantly found, which are also observed in the RHEED pattern oriented along the (11) in‐plane direction of the substrate, as shown in Figure 1d. Shortly after growth commenced, three distinct film streaks appeared corresponding to these two rotational domains observed in LEED. Due to a 30 ° rotation between domains, they are assigned to the (10) order for domain 1 (blue), the (11) order of domain 2 (orange), and (20) order of domain 1.

AFM has been employed to assess the surface morphology exemplified in Figure 1e for a sample without capping and a thickness of 9 nm, which exhibits a root mean square roughness (RMS) of 1.05 nm. This value aligns well with roughness measurements obtained via XRR (see Table S1 and Figure S2, Supporting Information), confirming consistency across methods used for thickness and roughness determination.

Figure 1f presents the 𝜃–2𝜃 diffractograms that confirm the high sample quality, as evidenced by the existence of distinct Laue fringes. These fringes arise from the interference of X‐rays reflected from the top and bottom interfaces of a crystalline film with high structural order, i.e., smooth surfaces and interfaces as well as a uniform crystallinity, which means a single, well‐oriented grain in the direction of the film normal. High‐resolution scanning electron microscopy (SEM) top‐view images are shown in Figure S3 (Supporting Information). Specifically, clear oscillating patterns emerge for the thicker samples’ (003) peaks. Apart from the substrate and those corresponding to (003) and (006) peaks, no additional peaks are observed. The intensity of the (006) peak is less than that of the (003) peak, in line with the calculated powder diffraction pattern (intensity ratio of 100 to 0.01, respectively).

Figure 1g presents rocking curves from the thin film samples, measured on the In_2_Te_3_(003) Bragg reflection. These curves exhibit distinct peaks with full‐width half‐maximum (FWHM) values of ≈ 0.1 °, corroborating the excellent texture and uniformity of the films across all thicknesses studied. Notably, as film thickness decreases, the peak positions shift slightly toward lower angles, an effect indicative of mechanical relaxation due to strain at the film, substrate interface and possible minor tilting of crystallites relative to the sample normal.

Pseudo–Voigt fits for both peaks have been used to determine the out‐of‐plane lattice constant *c*, using the average of the (003) and (006) results. For the thinnest sample, interference occurs between the (003) peak and the Si (111) peak. To address this, the silicon peak has been incorporated in the fit, as shown in Figure S4 (Supporting Information). For all remaining samples, contributions from silicon were not considered. The out‐of‐plane lattice constant of the thickest sample was determined to be *c* = 10.69 Å. The in‐plane lattice constant of the thickest sample was determined to be *a* = 4.38 Å based on RHEED data. Both experimental in‐plane and out‐of‐plane lattice constants show good agreement with literature values, with an increase of 0.2 % and 0.5 %, respectively.

**Figure 2:**
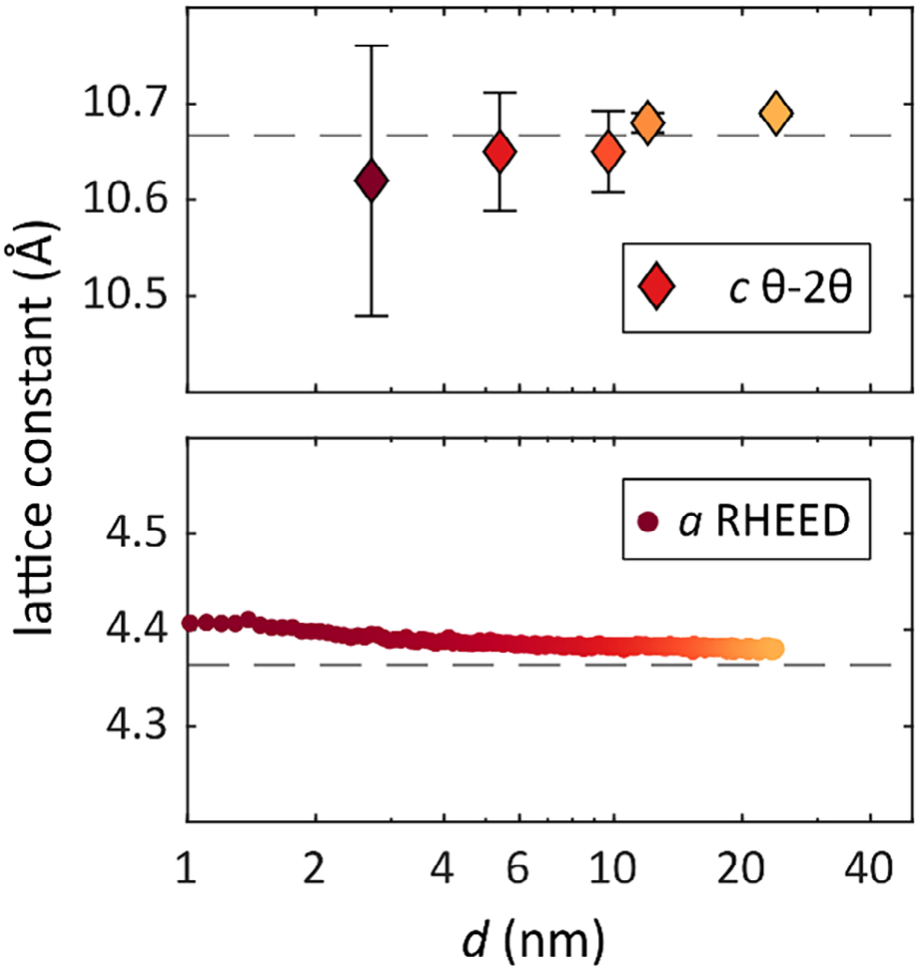
Thickness‐dependent lattice constants of In_2_Te_3_. The in‐plane lattice constant has been extracted from the (10) streaks in the RHEED growth video, which displays an increase of 0.4% between the thicknesses of 24 and 2.7 nm. The out‐of‐plane lattice constant has been extracted from the XRD spectra based on the average of (003) and (006) peaks. The literature bulk values are shown as dashed lines.^[^ ^31^ ^]^

**Figure** **2** illustrates how both lattice constants change as the film thickness decreases. The in‐plane lattice constant exhibits a slight increase of ≈0.02 Å (0.4 %) between the thicknesses of 24 and 2.7 nm, as shown in the lower part of Figure 2. During initial growth stages, strong overlap occurred between (11) and (20) RHEED streaks with those from the substrate; therefore, only (10) streaks were used to extract the in‐plane lattice constant.

Given the significant lattice mismatch between the Si (111) substrate and the In_2_Te_3_ film (3.86 Å versus 4.38 Å), which amounts to 11.9 %, it is surprising to observe an increase in the in‐plane lattice constant. Instead, one would expect it to decrease and converge toward 3.86 Å. However, when considering the film's in‐plane domain rotated by 30° relative to Si (111), a coincidence lattice can be identified, as shown in the supplementary information (Figure S5, Supporting Information). This adjustment reduces the mismatch to 1.8 % and explains the observed increase in the in‐plane lattice constant with decreasing film thickness as a result of strain from the substrate.

On the other hand, the out‐of‐plane lattice constant *c* shows a slight decrease with decreasing film thickness by ≈ 0.5 % between the thickest and thinnest films. This decrease correlates with the in‐plane increase, which effectively preservers the unit cell volume. Notably, both lattice parameters converge toward the bulk literature values for film thicknesses above 12 nm.

## 3 Dielectric Function of In_2_Te_3_ Thin Films and its Response to Thickness Reduction

**Figure 3:**
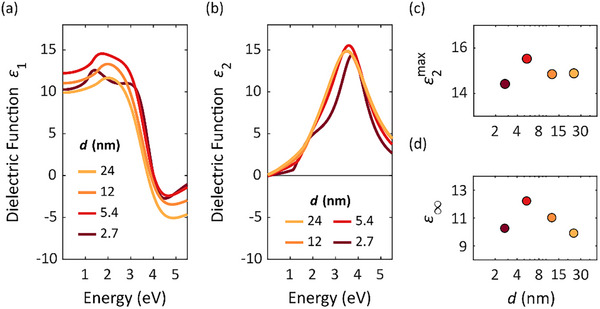
The real part a) and imaginary part b) of the dielectric function of four different In_2_Te_3_ thin films obtained by optical spectroscopy. c) Maximum value of ɛ_2_(ω) and the optical dielectric constant ɛ_∞_ d) plotted as a function of film thickness.

The consistent high structural quality throughout the range of film thicknesses is advantageous for the subsequent assessment of optical properties. To investigate the dielectric properties as a function of film thickness, the reflectance of the sample is recorded using Fourier‐Transform Infrared (FTIR) and fiber grating spectroscopy over a wide spectral range from far infrared to near ultraviolet (UV). The measured reflectance data are shown in Figure S6 (Supporting Information). This data is complemented by ellipsometry measurements from near infrared to UV. The dielectric function of In_2_Te_3_ was obtained by building a layer stack model and fitting the reflectance and ellipsometry data by using Tauc‐Lorentz oscillators. The resulting dielectric functions for different thicknesses are displayed in **Figure** **3**; the real part is shown in Figure 3a, and the imaginary part in Figure 3b.

The optical dielectric constant ɛ_∞_ has moderate values ranging from 10 to 13. As shown in Figure 3d, there is a subtle increase in ɛ_∞_ as the film thickness decreases down to 5.4 nm. However, for the thinnest sample (2.7 nm), ɛ_∞_ drops again toward the value observed in thicker films. Changes in dielectric properties with decreasing thickness have also been reported for other covalent chalcogenides. For example, calculations for GeSe reveal subtle variations in dielectric behavior as the material approaches ultrathin slab dimensions. This could indicate that In_2_Te_3_ likewise experiences changes in electron localization and structural distortion in its thinnest layers, although the effect appears to be much weaker than what is typically observed in metavalent chalcogenides. This optical dielectric constant ɛ_∞_ is defined at frequencies above the highest phonon resonance but below the next natural frequency of the crystal, i.e., the smallest possible interband transition (bandgap).^[^ ^32^ ^]^ To meet these criteria set forth in the definition, ɛ_∞_ values may be extracted at an energy of 0.05 eV.

The imaginary part of the dielectric function shows a distinct maximum associated with an interband transition at ≈ 3.5 eV. Importantly, no significant dependence on film thickness is observed. For all samples, 𝜀_2_^max^ is between 14 and 16 as shown in Figure 3c. Notably, the energy associated with the interband transition experiences a small shift of 0.3 eV toward higher energies upon reducing the layer thickness, transitioning from 3.5 to 3.8 eV, indicating a subtle change in absorption characteristics. To delve deeper into these phenomena, we will explore the thickness‐dependent light‐matter interactions and their implications for lattice dynamics in In_2_Te_3_ thin films in the subsequent section.

## 4 Lattice Dynamics of In_2_Te_3_ Thin Films and Their Response to Thickness Reduction

The previous sections have demonstrated that the atomic structure and optical properties depend only slightly on film thickness. Consequently, we expect that photoexcitation and the resulting lattice dynamics will also remain largely unchanged with varying film thicknesses. To investigate the dynamics of the charge carriers and the crystal lattice in response to an external stimulus, femtosecond pump‐probe measurements were conducted.

**Figure 4:**
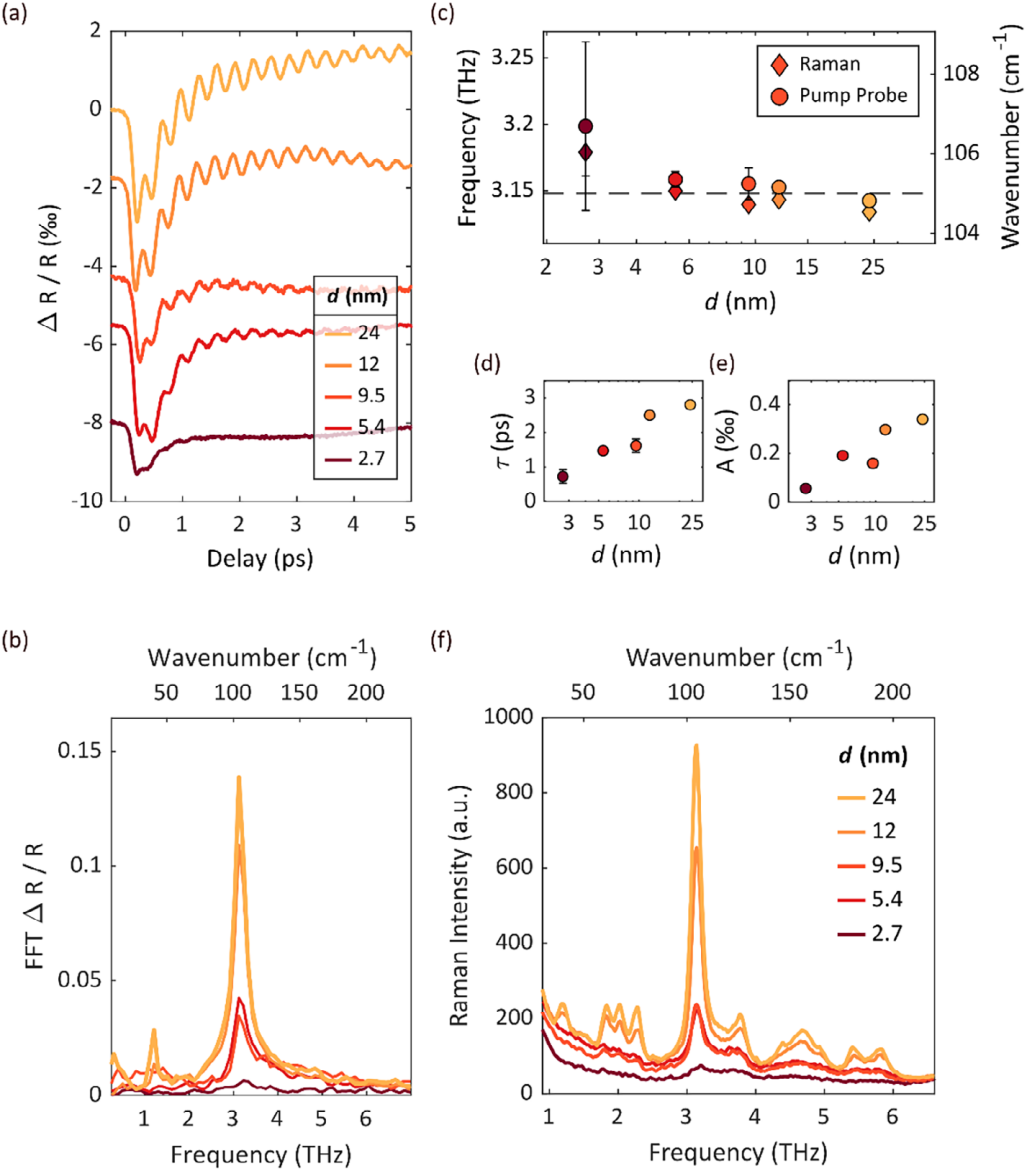
a) Isotropic transient reflectance (in per mille) for the thin film samples. Curves are vertically shifted for clarity. The excitation of coherent phonons is visible in each curve. b) Fourier transformation of the oscillatory component of the signal showing two distinct phonon frequencies. The mode frequencies resulting from damped harmonic oscillator fits are shown in c). The corresponding decoherence times and amplitudes against the film thickness are in d,e). f) Raman Shift data for different film thicknesses. Peak positions from Raman spectroscopy data are shown as diamonds in (c).

**Figure** **4**a displays the isotropic transient reflectance for films of different thicknesses, with curves vertically shifted for clarity. The initial change in reflectance observed within the first few hundred femtoseconds is due to the direct response of the excited carriers and the formation of non‐equilibrium excited states.^[^ ^33^ ^]^ This excess energy is partially converted into coherent optical phonons, which can be observed for all film thicknesses.^[^ ^33^, ^34^, ^35^ ^]^

To determine the frequencies of these coherent phonons, we first subtract the background using a phenomenological model that describes the initial excitation of carriers and their subsequent relaxation. The oscillatory part of the signals can be Fourier transformed to extract phonon frequencies, illustrated in Figure 4b.

A dominant peak at ≈3.15 THz (105 cm^−1^) is observed for all film thicknesses, while a smaller peak at 1.2 THz (40 cm^−1^) is present only in the two thickest films. These modes align well with the Raman literature values at 105 and 42 cm^−1^,^[^ ^36^, ^37^ ^]^ where the phonon mode at 105 cm^−1^ has been identified as an *A*_1g_ symmetry phonon.^[^ ^37^ ^]^ This observation corresponds well with its dominance in isotropic transient reflectance measurements, which primarily resolve fully symmetric *A*_1g_ phonons.^[^ ^35^ ^]^

A more accurate understanding is achieved by fitting these oscillations with damped harmonic oscillators. The explicit plots of our fitting curves overlaid on the background‐subtracted transient reflectance data for all film thicknesses are provided in the Supporting Information (Figure S7, Supporting Information). The resulting frequencies show a slight increase (≈ 1 %) with decreasing film thickness, as depicted in Figure 4c. Both decoherence time *τ* and amplitude *A* of the reflectance changes generated by coherent phonons decrease as film thickness diminishes. This trend is illustrated in Figure 4d,e. Specifically, decoherence time decreases from 3 ps to below 1 ps, while amplitude drops from 0.4 ‰ to 0.08 ‰. Although the coherent phonon oscillation is less pronounced in the 2.7 nm film compared to thicker samples, its presence can still be identified. For enhanced clarity, we provide a detailed analysis of the coherent phonon oscillations for the 2.7 nm film in the Supporting Information (Figure S8, Supporting Information).

Additionally, Raman spectroscopy provides insights into vibrational properties without optical pre‐excitation. The corresponding spectra are presented in Figure 4f. The phonon peak at 105 cm^−1^ dominates the spectrum across all layer thicknesses; however, the previously noted peak at 40 cm^−1^ was resolved only for the two thickest samples. By employing Lorentzian fits, we determined peak positions, which are compared to the pump‐probe data shown in Figure 4c, exhibiting good agreement. It should be noted that we identified a total of 11 distinct phonon peaks in the data from the thickest sample, which align well with existing literature on bulk samples.^[^ ^36^ ^]^ This is further discussed in the Supporting Information (Figure S9, Supporting Information).

## 5 Discussion

The results presented demonstrate that thin In_2_Te_3_ films grown on Si (111) exhibit only minor dependency on thickness. Specifically, a relative change of below 1 % is observed in both lattice parameters. Furthermore, no substantial shifts in the dielectric constant or the height of the absorption maximum are reported. The frequency of the dominant *A*_1g_ phonon mode increased moderately by ≈ 1 %.

This behavior contrasts sharply with that of other sesqui‐chalcogenides, monochalcogenides, and pnictogens.^[^ ^21^, ^22^ ^]^ For instance, Sb_2_Te_3_, GeTe, and Bi show a relative change exceeding 5 % in the frequency of their *A*_1g_ phonon modes with comparable reductions in film thickness.^[^ ^19^, ^20^, ^22^ ^]^ In these materials, the out‐of‐plane lattice constant *c* shows a pronounced dependency on sample thickness and increases for thinner films, contrary to the results presented here. Additionally, the dielectric functions of these materials change significantly; both the dielectric constant and the height of the absorption maximum are reduced by half. Concurrently, the position of the absorption maximum shifts toward higher photon energies.^[^ ^21^ ^]^

The distinct behavior of In_2_Te_3_ can be closely linked to its chemical bonding mechanisms, different from other sesqui‐chalcogenides. Several bond indicators support this observation: a low Effective Coordination Number (ECoN) of 4; a low mode‐specific Grüneisen parameter ranging from 1.5–2.1;^[^ ^38^ ^]^ and a room temperature conductivity measured at ≈10^−1^ S cm^−1^,^[^ ^38^ ^]^ all suggesting covalent bonding in In_2_Te_3_. The low values shown for its dielectric function in Figure 3 further support this characterization as they align with those found in other covalently bonded semiconductors.

Two quantum‐chemical bond descriptors, electrons shared (ES) and electrons transferred (ET), are introduced to characterize electron distribution while varying the arrangement of vacancies.^[^ ^39^, ^40^ ^]^ As shown in Figure S10 (Supporting Information), the ES values of all structures range from 1.6 to 2.2, accompanied by moderate ET values (<0.25). These bonding descriptors fall within the range characteristic of covalent bonding, regardless of the specific vacancy distribution.^[^ ^39^, ^40^ ^]^

In contrast, Sb_2_Te_3_, GeTe, and Bi exhibit a different bonding mechanism characterized by a competition between electron localization and delocalization due to a network of overlapping *p*‐orbitals. This results in a unique conductivity range (between 10^3^ and 10^4^ S cm^−1^) that situates these materials between semiconductors and metals, earning them the designation “incipient metals.”^[^ ^25^ ^]^ These incipient metals share a common characteristic: their chemical bonding between atoms is primarily influenced by half‐filled *p*‐orbitals.^[^ ^24^, ^25^ ^]^ In reduced dimensions, the competition between electron localization and delocalization is modified, and an increased degree of electron localization alters material properties and crystal structures.^[^ ^21^, ^23^ ^]^

For metavalent materials, out‐of‐plane relaxation occurs through a mechanism where electron localization and delocalization adjust upon changing film thickness to minimize energy by altering atomic positions.^[^ ^21^, ^22^ ^]^ This mechanism has been documented extensively for thin GeTe layers.^[^ ^41^ ^]^ Importantly, the metavalently bonded thin films expand their out‐of‐plane lattice constant by several percent upon reduction in thickness; in In_2_Te_3,_ it reduces.

Structural distortions within thin films due to variations in bond strengths significantly influence phonon mode frequencies. For layered chalcogenides with n‐tuple stacking along the (001) direction ‐ specifically Sb_2_Te_3_ ‐ the *A*_1g_^1^ mode exhibits a redshift while the *A*_1g_^2^ mode shows a blueshift. Consequently, no strong trend emerges regarding phonon frequencies in In_2_Te_3_ (as shown in Figure 4), since significant changes do not occur within chemical bonding and thus atomic structure when layer thickness is reduced.

## 6 Conclusion

In summary, this study has examined the impact of thickness on the properties of thin In_2_Te_3_ films. The results demonstrate that the intrinsic material properties of covalent In_2_Te_3_ films remain nearly constant even when the film thickness is drastically reduced. This behavior contrasts sharply with that of metavalent sesqui‐chalcogenides such as Sb_2_Te_3_, Bi_2_Te_3_, or Bi_2_Se_3_, which exhibit significant changes in bonding and intrinsic properties upon reduction in film thickness. Only small changes were observed in the structural, optical, and vibrational properties of In_2_Te_3_ films. This finding suggests that other covalent sesqui‐chalcogenides such as Bi_2_S_3_, Sb_2_Se_3_, and Sb_2_S_3_ may also experience only modest changes in their intrinsic material properties when film thickness is reduced. Despite differences in crystal structure among these materials, they share similar chemical bonding characteristics with In_2_Te_3_.

## 7 Experimental Section

### Growth

Prior to insertion into the UHV chamber, the substrates (Okmentic, Si(111), SSP, p doped, > 5 kΩ) underwent a cleaning process with H_2_SO_4_:H_2_O_2_. Subsequently, they were immersed in HF to remove the native oxide layer and passivate the dangling bonds with hydrogen.

The substrates were heated to T = 330 °C in UHV to evaporate the hydrogen and subsequently cooled down to the growth temperature of T = 210 °C. The temperature of 330 °C is shown to preserve the 1 × 1 Si(111) surface, while ensuring the evaporation of the hydrogen passivation layer in the growth chamber. The optimal growth temperature of 210 °C was determined through systematic variation of the deposition temperature. Note that all temperature readings have an offset due to the thermocouple reading. The unreconstructed surface was validated using RHEED. Optimal growth conditions for growth along the [111] orientation were determined in a tellurium‐rich environment with a beam flux ratio In: Te of 1:20. Both elements were evaporated from standard effusion cells. The growth rate was 0.1 nm min^−1^, monitored by RHEED. After growth, the samples were capped in situ with an Al_2_O_3_ layer with a thickness of ≈10 nm, employing sputtering.

### LEED

After growth, the sample has been in situ transferred to the LEED chamber, where the LEED pattern was acquired with a Specs ErLEED 1000‐A Optics and a background pressure of 1 × 10^−10^ mbar.

### AFM

AFM measurements were performed for a sample without capping using a Bruker Dimension Edge AFM. The scan size was 10 µm × 10 µm, and 512 × 512 scans were taken with a tapping frequency of 0.5 Hz.

### X‐Ray Reflection and Diffraction

The measurements were measured on a “Rigaku Smartlab” system with a rotating anode employing a Cu Kα1 radiation source (λ = 1.54 Å), selected by a Ge (220) 2 bounce monochromator.

### Femtosecond Pump Probe Reflectivity

Ultrafast optical measurements were conducted using a standard reflection‐type, two‐color pump‐probe experiment in an isotropic configuration. The 800 nm wavelength, 60 fs width pump beam was separated from a Ti:Sapphire regenerative femtosecond amplifier, chopped at 1500 Hz, and directed to a free‐standing optical delay line before being focused on the sample to a spot size of 200 µm in diameter. The probe pulses were frequency converted to 516 nm via sum frequency generation in an optical parametric amplifier and focused to a 30 µm diameter spot on the sample. The detection unit comprised two balanced Si photodiodes connected to variable‐gain current amplifiers and a data acquisition card. All measurements were taken at incident pump fluences of ≈1 mJ cm^−^^2^, with the probe fluence being ten times lower. To eliminate systematic errors on laboratory time scales, the order of data point recording and the positioning of the delay line were randomized. The isotropic transient reflectance was calculated from the whole signal:

(1) $$\Delta R=\frac{\Delta \left(R_{s}+R_{p}\right)}{R_{0}}$$

A polarizing beam splitter cube was used to split the reflected probe beam correspondingly. Transient reflectance was normalized to the steady state reflectance *R*_0_. Reversibility of optical excitation was ensured by monitoring the static reflectivity gained for the probe‐pulse when the pump‐pulse was chopped.

### Raman Spectroscopy

Raman spectroscopy measurements were conducted with a commercial WITec system (alpha300), operating in backscattering geometry. A diode‐pumped solid‐state laser supplied linearly polarized light with a wavelength of 531 nm (2.33 eV). This was delivered through a single‐mode optical fiber and used to excite the sample. The spot size of 400–500 nm was obtained by employing a long working focusing lens with a numerical aperture of 0.70. By setting the excitation power to 500 µW, significant heating effects were avoided. For detection, the reflected light was delivered through a single‐mode optical fiber to a charge‐coupled spectrometer with a grating of 2400 lines per mm. All measurements were obtained at room temperature with a 50× (NA = 0.7) objective, with a resolution close to the diffraction limit (≈500 nm).

### Optical Spectroscopy

Ellipsometry spectra were measured using three angles of incidence (65°, 70°, and 75°) on a J.A.Woollam M‐2000UI spectroscopic ellipsometer. The deuterium and halogen lamps served as the sources of illumination for the setup. A silicon CCD camera detected visible and UV light, while an InGaAs diode array captured lower‐energy photons. In total, 584 channels with an average of 7 meV resolution over 0.72 to 5.14 eV were available. Normal incidence reflectance data were collected by fiber grating in the range from 9100 to 45 000 cm^−1^ with an Avantes AvaSpec‐ULS2048CL‐EVO with the use of a CMOS linear image sensor. Deuterium and halogen lamps (AvaLight‐DH‐S‐BAL) were used as illumination sources. An aluminum mirror served as a reference. Reflectance measurements in the 200 respectively 100 to 18 000 cm^−1^ range were performed on a Bruker Vertex 80v Fourier‐transform infrared spectrometer (FTIR). A 200 nm thick silver mirror served as a reference. Multiple sources (Hg‐arc, globar, and tungsten lamp), beam splitter (Mylar 50 µm, multilayer, KBr, and CaF2), and detector (DLaTGS (one with polyethylene and one with KBr window), liquid nitrogen‐cooled InSb, and room‐temperature Si photodiode) combinations were utilized to cover the entire spectral range. The dielectric functions of the samples were determined using a five‐layer model within the J.A. Woollam CompleteEASE 6 software. The bottom layer consists of the silicon substrate, followed by the indium telluride thin film. The dielectric function of the thin film was described by employing a summation of two Tauc‐Lorentz oscillators without a Drude contribution. The top two layers represent the capping in an effective medium approach (EMA) to account for a mixture of aluminum oxide with and without a slight amount of iron impurity, both of which were measured independently in advance. An EMA layer describing a roughness layer obtained from X‐ray reflectivity (XRR) measurements is situated between the capping layers and the thin film. All EMA layers were fitted using the Bruggeman model, and all parameters were adjusted until convergence was achieved.

### DFT Studies

To validate these experimental observations, density functional theory (DFT) were carried out using the Vienna Ab Initio Simulation Package (VASP) with the projector‐augmented wave pseudopotentials.^[^ ^42^, ^43^, ^44^, ^45^ ^]^ The Perdew–Burke–Ernzerhof functional is employed to approximate the exchange−correlation energies of electrons.^[^ ^46^ ^]^ The cutoff energies for the plane‐wave basis were set to 550 eV. The quantification of electrons shared (ES) within chemical bonding utilizes the density‐derived electrostatic and chemical (DDEC) approach.^[^ ^47^, ^48^, ^49^ ^]^ ES was quantified as twice the value of the bond order.

### Scanning Electron Microscopy

Scanning Electron Microscopy was performed using a FEI Nanolab 650. The morphology of the films was imaged using an electron voltage of 20 kV and an electron current of 1.6 nA in the immersion mode and working distance of 4.2 mm. The electrons were detected via a TLD detector. Additionally, cross‐section images were performed by depositing a protective layer of Pt via the gas injection system and milling perpendicular to the sample surface with the Ga^+^‐FIB into the sample. For the latter, a cross‐section pattern was used. Deposition and milling steps were performed using 30 kV and 80 pA. Subsequently, the layer stack was imaged again in the immersion mode with 20 kV electron voltage and 1.6 nA at 52° stage tilt.

### Proofreading

OpenAI GPT 4o was used to check grammar, syntax, and clarity. The content and scientific accuracy of the paper remain the responsibility of the authors.

## Conflict of Interest

The authors declare no conflict of interest.

## Author Contributions

M.B. and F.H. contributed equally to this work. M.B. initiated the project. M.W. supervised the project. M.B., C.R., K.L.M., and L.B. fabricated the samples and performed XRD, XRR, RHEED, LEED, and AFM measurements and analyzed them. T.S. and N.P. conducted optical spectroscopy measurements and data analysis. J.K. performed SEM measurements. T.V., J.F., and F.H. performed pump probe and Raman measurements and analyzed the data. D.K. calculated ES values. F.H. prepared the figures. M.B. and F.H. wrote the manuscript. M.W. significantly revised the manuscript. All authors commented on and approved the submission of this manuscript.

## Supporting information



Supporting Information

## Data Availability

The data that support the findings of this study are available from the corresponding author upon reasonable request.

## References

[1] G. J. Snyder , E. S. Toberer , Nat. Mater. 2008, 7, 105. 18219332 10.1038/nmat2090

[2] D. A. Wright , Nature 1958, 181, 834.

[3] S. Raoux , W. Wełnic , D. Ielmini , Chem. Rev. 2010, 110, 240. 19715293 10.1021/cr900040x

[4] M. Wuttig , N. Yamada , Nat. Mater. 2007, 6, 824. 17972937 10.1038/nmat2009

[5] H. Zhang , C.‐X. Liu , X.‐L. Qi , X. Dai , Z. Fang , S.‐C. Zhang , Nat. Phys. 2009, 5, 438.

[6] Y. Chen , J. G. Analytis , J.‐H. Chu , Z. Liu , S.‐K. Mo , X.‐L. Qi , H. Zhang , D. Lu , X. Dai , Z. Fang , Science 2009, 325, 178. 19520912 10.1126/science.1173034

[7] W. Li , X.‐F. Cai , N. Valdes , T. Wang , W. Shafarman , S.‐H. Wei , A. Janotti , J. Phys. Chem. Lett. 2022, 13, 12026. 36541824 10.1021/acs.jpclett.2c02975

[8] Y.‐T. Huang , N.‐K. Chen , Z.‐Z. Li , X.‐B. Li , X.‐P. Wang , Q.‐D. Chen , H.‐B. Sun , S. Zhang , Appl. Phys. Rev. 2021, 8, 031413.

[9] Y. T. Huang , N. K. Chen , Z. Z. Li , X. P. Wang , H. B. Sun , S. Zhang , X. B. Li , InfoMat 2022, 4, 12341.

[10] S. R. Tamalampudi , Y.‐Y. Lu , U. Rajesh Kumar , R. Sankar , C.‐D. Liao , C.‐H. Cheng , F. C. Chou , Y.‐T. Chen , Nano Lett. 2014, 14, 2800. 24742243 10.1021/nl500817g

[11] I. Kim , J. Ryu , E. Lee , S. Lee , S. Lee , W. Suh , J. Lee , M. Kim , H. Oh , G.‐C. Yi , NPG Asia Mater. 2024, 16, 59.

[12] Y. Huangfu , B. Qin , P. Lu , Q. Zhang , W. Li , J. Liang , Z. Liang , J. Liu , M. Liu , X. Lin , Small 2024, 20, 2309620. 10.1002/smll.202309620 38294996

[13] J. Yao , Z. Deng , Z. Zheng , G. Yang , ACS Appl. Mater. Interfaces 2016, 8, 20872. 27459243 10.1021/acsami.6b06222

[14] M. H. Ali , M. A. Al Mamun , M. D. Haque , M. F. Rahman , M. K. Hossain , A. Z. Md , ACS Omega 2023, 8, 7017. 36844558 10.1021/acsomega.2c07846 PMC9948157

[15] H. Hahn , W. Klingler , Z. für Anorg. Chem. 1949, 260, 97.

[16] J. Woolley , B. Pamplin , P. Holmes , J. Less‐Common Met. 1959, 1, 362.

[17] T. Karakostas , N. A. Economou , Phys. Status Solidi A 1975, 31, 89.

[18] S. Zhang , J. Zhang , B. Liu , X. Jia , G. Wang , H. Chang , Sci. Rep. 2019, 9, 10951. 31358867 10.1038/s41598-019-47520-x PMC6662755

[19] P. Kerres , Y. Zhou , H. Vaishnav , M. Raghuwanshi , J. Wang , M. Häser , M. Pohlmann , Y. Cheng , C. F. Schön , T. Jansen , Small 2022, 18, 2201753. 10.1002/smll.202201753 35491494

[20] J. Mertens , P. Kerres , Y. Xu , M. Raghuwanshi , D. Kim , C. F. Schön , J. Frank , F. Hoff , Y. Zhou , R. Mazzarello , Adv. Funct. Mater. 2024, 34, 2307681.

[21] P. Kerres , R. Mazzarello , O. Cojocaru‐Mirédin , M. Wuttig , Phys. Status Solidi A 2024, 221, 2300921.

[22] F. Hoff , P. Kerres , T. Veslin , A. R. Jalil , T. Schmidt , S. Ritarossi , J. Köttgen , L. Bothe , J. Frank , C. F. Schön , Adv. Mater. 2025, 37, 2416938. 39740119 10.1002/adma.202416938 PMC11837888

[23] B. J. Kooi , M. Wuttig , Adv. Mater. 2020, 32, 1908302. 10.1002/adma.201908302 32243014

[24] M. Wuttig , C. F. Schön , J. Lötfering , P. Golub , C. Gatti , J. Y. Raty , Adv. Mater. 2023, 35, 2208485. 10.1002/adma.202208485 36456187

[25] M. Wuttig , V. L. Deringer , X. Gonze , C. Bichara , J. Y. Raty , Adv. Mater. 2018, 30, 1803777. 10.1002/adma.201803777 30318844

[26] O. Cojocaru‐Mirédin , Y. Yu , J. Köttgen , T. Ghosh , C. F. Schön , S. Han , C. Zhou , M. Zhu , M. Wuttig , Adv. Mater. 2024, 36, 2403046. 39520347 10.1002/adma.202403046 PMC11636162

[27] S. Maier , S. Steinberg , Y. Cheng , C. F. Schön , M. Schumacher , R. Mazzarello , P. Golub , R. Nelson , O. Cojocaru‐Mirédin , J. Y. Raty , Adv. Mater. 2020, 32, 2005533. 10.1002/adma.202005533 33135228

[28] S. Lee , K. Esfarjani , T. Luo , J. Zhou , Z. Tian , G. Chen , Nat. Commun. 2014, 5, 3525. 24770354 10.1038/ncomms4525

[29] K. Shportko , S. Kremers , M. Woda , D. Lencer , J. Robertson , M. Wuttig , Nat. Mater. 2008, 7, 653. 18622406 10.1038/nmat2226

[30] J. Hogg , H. Sutherland , Struct. Sci. 1976, 32, 2689.

[31] V. Zhuze , A. Zaslavskii , V. Petrusevich , Proc. Int. Conf. Phys. Semicond. 1960, 781.

[32] M. Fox , Optical Properties of Solids, Vol. 3, Oxford University Press, Oxford, England 2010.

[33] A. Othonos , J. Appl. Phys. 1998, 83, 1789.

[34] T. Cheng , J. Vidal , H. Zeiger , G. Dresselhaus , M. Dresselhaus , E. Ippen , Appl. Phys. Lett. 1991, 59, 1923.

[35] H. Zeiger , J. Vidal , T. Cheng , E. Ippen , G. Dresselhaus , M. Dresselhaus , Phys. Rev. B 1992, 45, 768. 10.1103/physrevb.45.768 10001117

[36] E. Finkman , J. Tauc , R. Kershaw , A. Wold , Phys. Rev. B 1975, 11, 3785.

[37] J. Zhen , W. Deng , C. Li , J. Feng , S. Zhang , S. Wan , G. Wang , H. Dong , R. A. Susilo , B. Chen , J. Phys. Chem. Lett. 2022, 13, 1226. 35089034 10.1021/acs.jpclett.1c04124

[38] E. Zuñiga‐Puelles , A. Özden , R. Cardoso‐Gil , C. Hennig , C. Himcinschi , J. Kortus , R. Gumeniuk , J. Mater. Chem. A 2025, 9357. 10.1039/c9dt01902b 31243411

[39] J.‐Y. Raty , M. Wuttig , J. Phys. D: Appl. Phys. 2020, 53, 234002.

[40] J. Y. Raty , M. Schumacher , P. Golub , V. L. Deringer , C. Gatti , M. Wuttig , Adv. Mater. 2019, 31, 1806280. 10.1002/adma.201806280 30474156

[41] R. Wang , W. Zhang , J. Momand , I. Ronneberger , J. E. Boschker , R. Mazzarello , B. J. Kooi , H. Riechert , M. Wuttig , R. Calarco , NPG Asia Mater. 2017, 9, 396.

[42] G. Kresse , J. Hafner , Phys. Rev. B 1993, 47, 558. 10.1103/physrevb.47.558 10004490

[43] G. Kresse , J. Furthmüller , Comput. Mater. Sci. 1996, 6, 15.

[44] G. Kresse , J. Furthmüller , Phys. Rev. B 1996, 54, 11169. 10.1103/physrevb.54.11169 9984901

[45] P. E. Blöchl , Phys. Rev. B 1994, 50, 17953. 10.1103/physrevb.50.17953 9976227

[46] J. P. Perdew , K. Burke , M. Ernzerhof , Phys. Rev. Lett. 1996, 77, 3865. 10062328 10.1103/PhysRevLett.77.3865

[47] T. A. Manz , N. G. Limas , RSC Adv. 2016, 6, 47771.

[48] N. G. Limas , T. A. Manz , RSC Adv. 2016, 6, 45727.

[49] T. A. Manz , RSC Adv. 2017, 7, 45552.

