# Supplementary material for: Robust Material Properties in Epitaxial In2Te3 Thin Films across Varying Thicknesses
Source: Small. 2025 Nov 14;21(51):e08738. doi: 10.1002/smll.202508738 (PMC12723348; doi:10.1002/smll.202508738)
Supplement: Supplementary file 1 — Supporting Information [file SMLL-21-e08738-s001.docx]

**Supporting Information**

**Robust Material Properties in Epitaxial In_2_Te_3_ Thin Films Across Varying Thicknesses**

Maximilian Buchta, Felix Hoff*, Lucas Bothe, Niklas Penner, Christoph Ringkamp, Thomas Schmidt, Jan Köttgen, Timo Veslin, Ka Lei Mak, Jonathan Frank, Dasol Kim and Matthias Wuttig

M. Buchta, L. Bothe, K. L. Mak, Prof. M. Wuttig

Peter-Grünberg-Institute – JARA-Institute Energy Eﬃcient Information Technology (PGI-10) Wilhelm-Johnen-Straße, 52428 Jülich, Germany

F. Hoff, N. Penner, C. Ringkamp, T. Schmidt, T. Veslin, J. Frank, D. Kim, Prof. M. Wuttig

Institute of Physics IA, RWTH Aachen University, Sommerfeldstraße, 52074 Aachen, Germany

E-Mail: [hoff@physik.rwth-aachen.de](mailto:hoff@physik.rwth-aachen.de)

Keywords: chalcogenides, optical properties, coherent phonons, molecular beam epitaxy, metavalent bonding


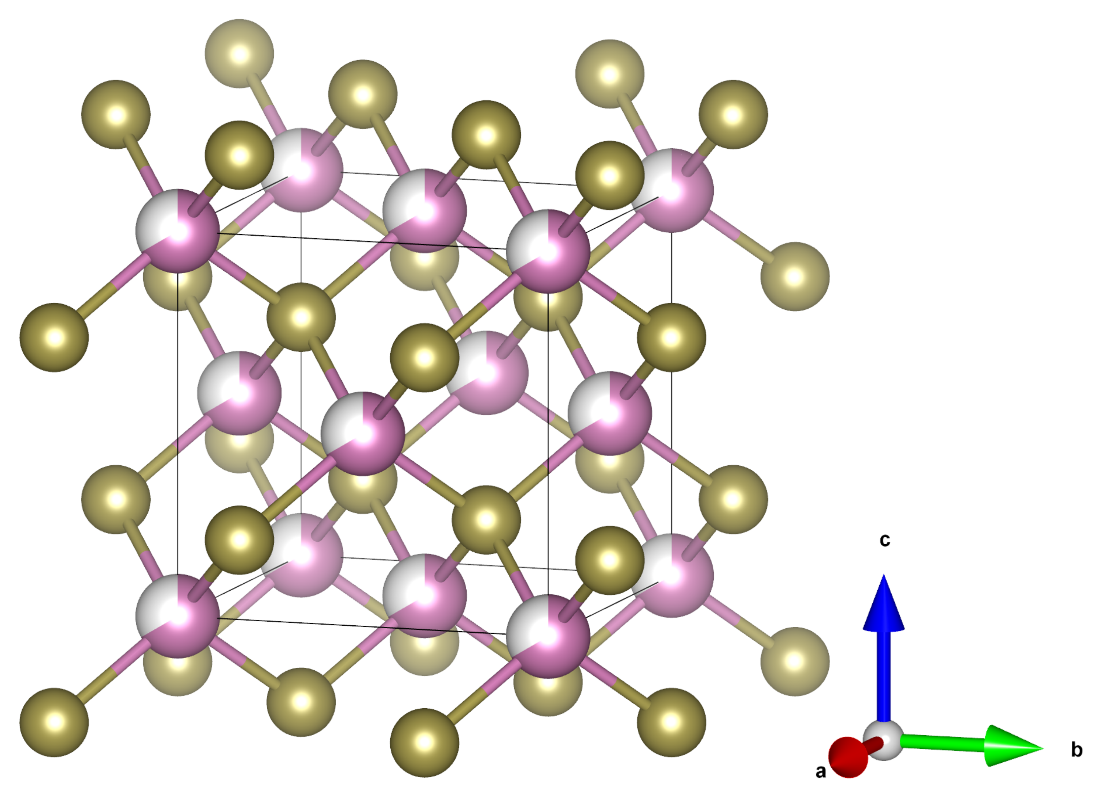


**Figure S1**: In_2_Te_3_ adopts the ZnS crystal structure [31], belonging to space group $F\overline{4}3m$. To compensate for the additional electron contributed by indium (displayed by the pink atoms) compared to zinc, the indium site is vacant by one third, homogenously distributed throughout the crystal.


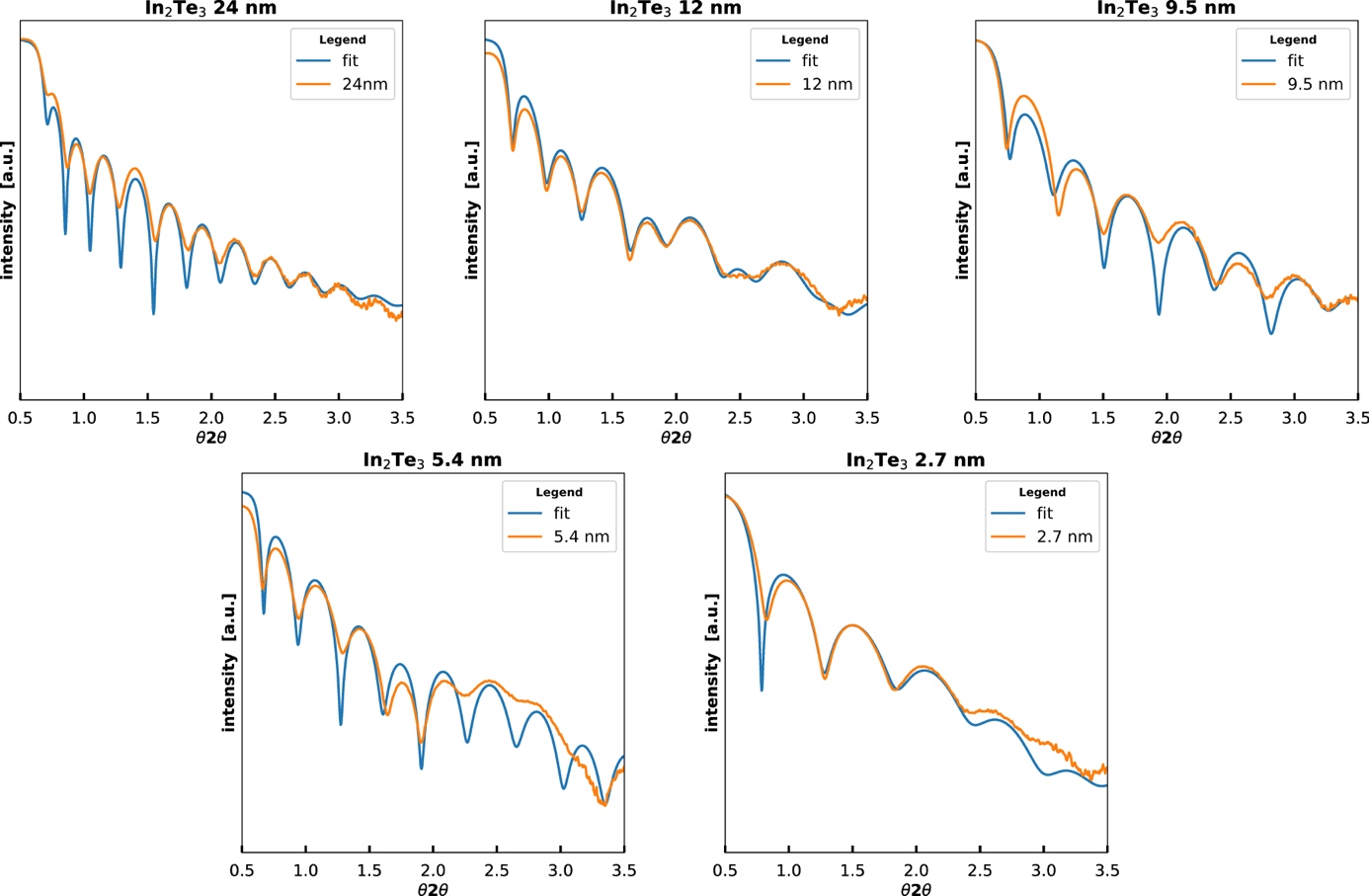


**Figure S2:** XRR measurements (orange curves) and fitted models (blue curves) for the In2Te3 thin films of varying thickness.

**Table S1:** Growth parameters and structural properties of In_2_Te_3_ thin films. Listed are the growth time, film thickness (from XRR), RMS roughness (from XRR), and the relative RMS roughness (RMS roughness divided by thickness) for each sample.

| **growth time[min]** | **Thickness [nm]** | **RMS roughness [nm]** | **Rel. RMS []** |
| --- | --- | --- | --- |
| **30** | **2.7** | **0.32** | **0.12** |
| **60** | **5.4** | **0.48** | **0.09** |
| **80** | **9.5** | **0.75** | **0.08** |
| **120** | **12.0** | **1.5** | **0.13** |
| **240** | **24.1** | **2.0** | **0.08** |


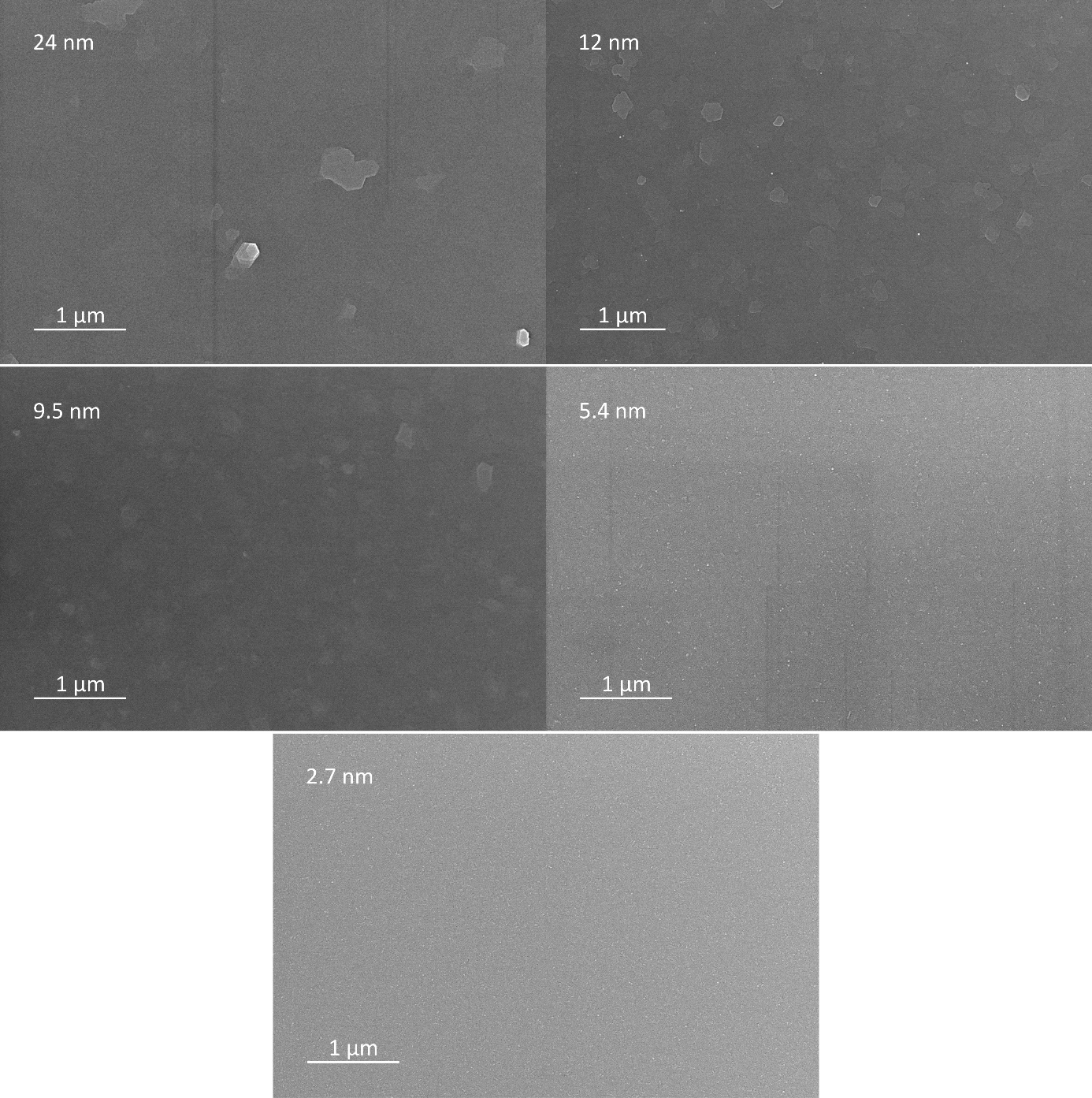


**Figure S3**: SEM top view of all films. Using an emission voltage of 20 kV and an electron current of 1.6 nA the surfaces of the samples were imaged in the immersion mode of the FEI Nanolab 650. By displaying the sample at a high magnification, the SEM images show clearly that the thin films were grown in a closed and smooth manner. The gray/black stripes visible in the images of the 24 nm and 5.4 nm films are measurement artifacts from the SEM and do not represent features of the sample surface.


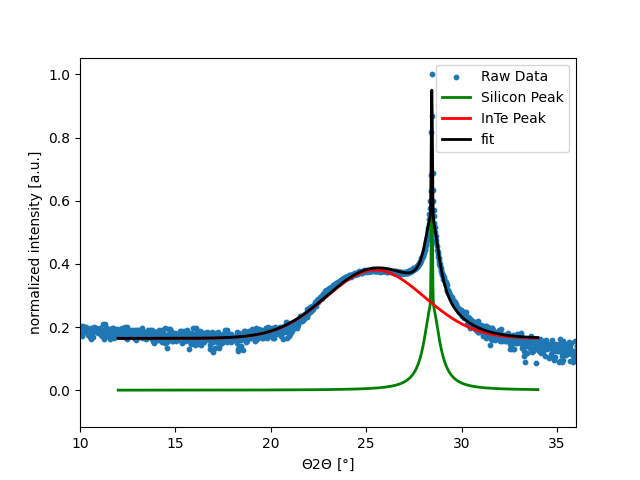


**Figure S4:** Fit of the In_2_Te_3_(003) Bragg peak for the 2.7 nm film. In this film, the substrate and film peaks overlap strongly and the substrate peak needed to be incorporated into the fit (green).


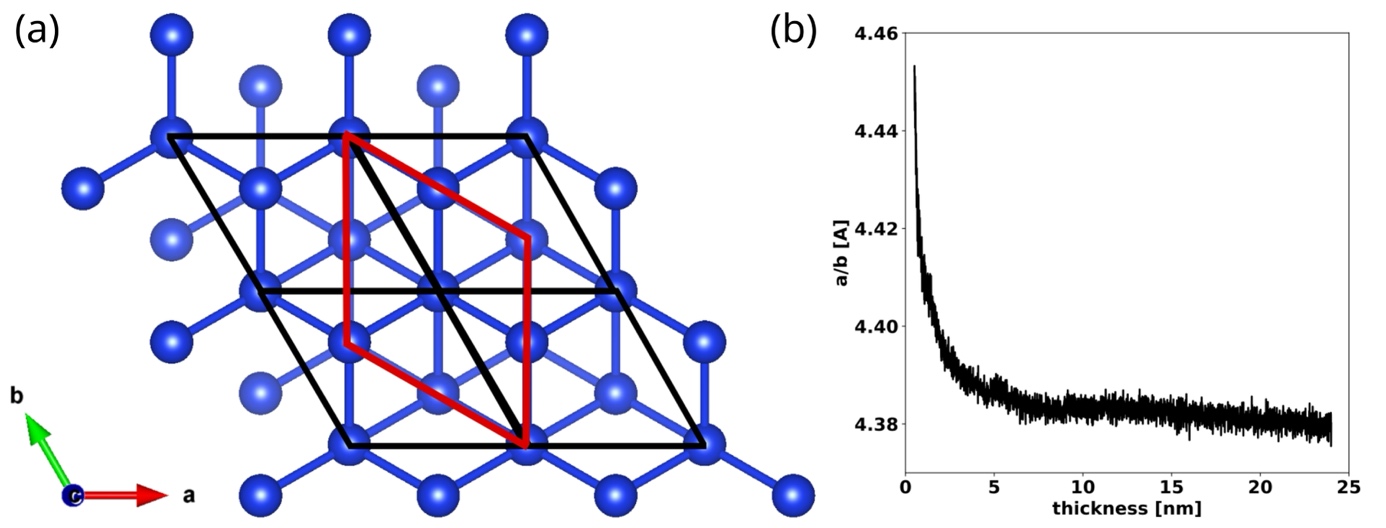


**Figure S5**: (**a**) Coincidence lattice of In_2_Te_3_(111) (red) and Si(111) (unit cell in black and atoms in blue) with a coincidence lattice constant of a = 4.46 Å, corresponding to a strain of 2.4 %. This alignment helps minimize strain at the interface between the substrate and the thin film. (**b**) RHEED data showing the evolution of the in-plane lattice constant during growth. Initially, the in-plane lattice constant is approximately 4.45 Å, indicating that the growth starts under strain towards the coincidence site lattice. This value sharply decreases within the first 2 nm of film thickness, after which the lattice constant gradually relaxes and converges towards the bulk value of In_2_Te_3_ as thickness increases.


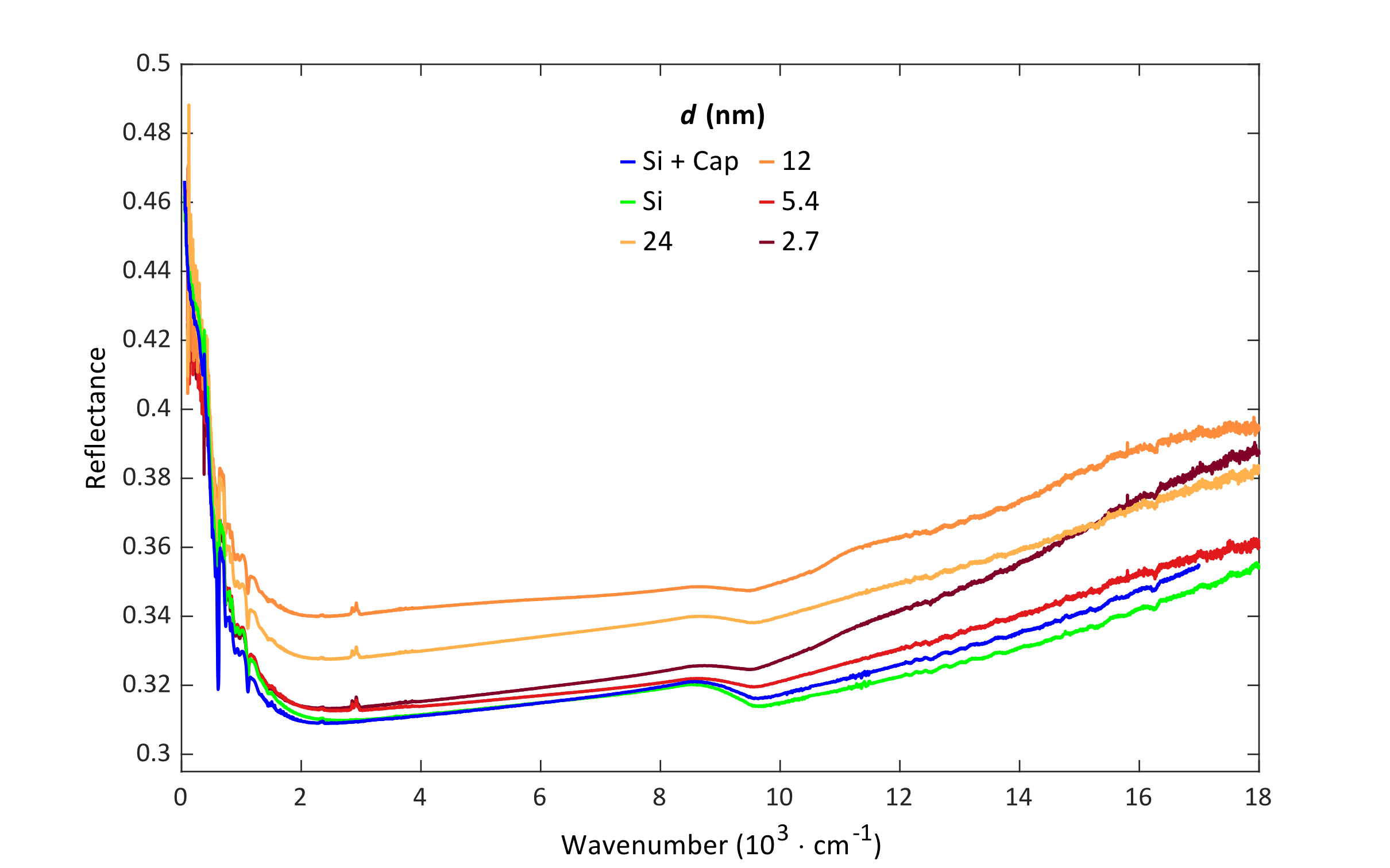


**Figure S6**: Reflectance raw data of the films of different thicknesses as well as bare Si substrate (green) and Si substrate with capping (blue).


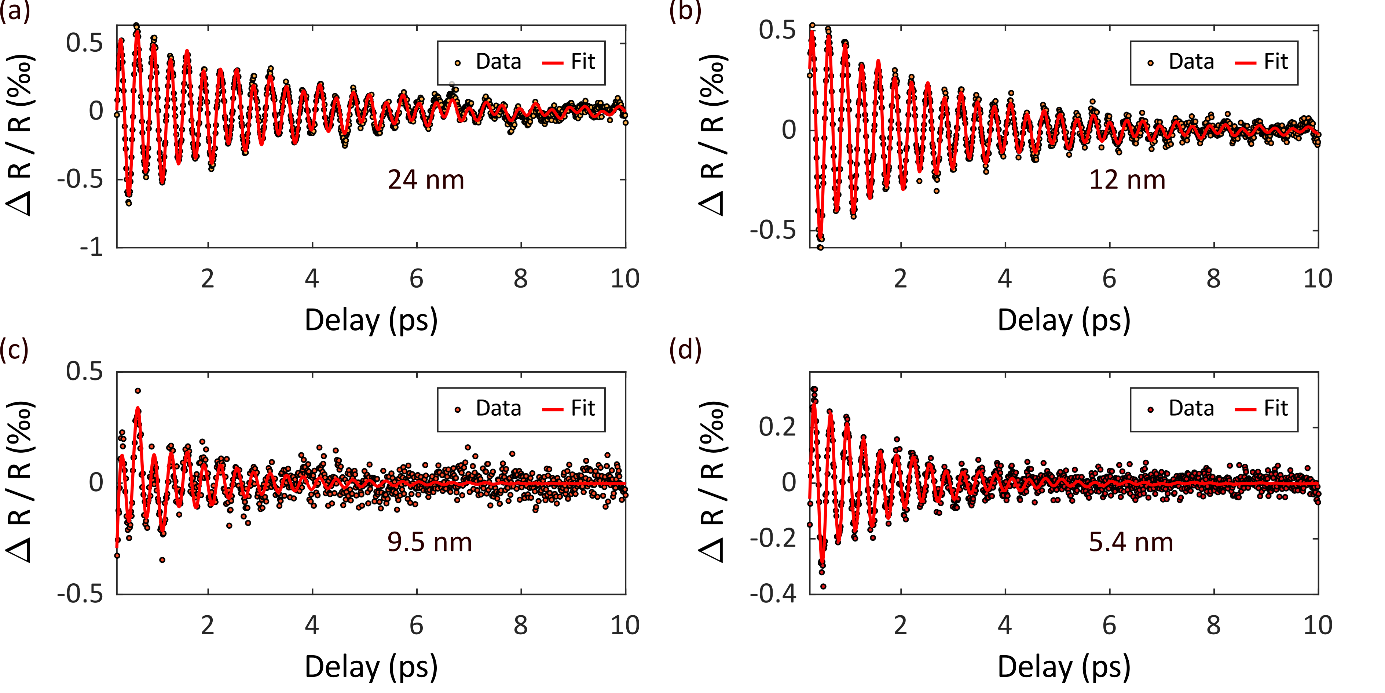


**Figure S7**: Damped harmonic oscillator fits (red lines) overlaid on background-subtracted transient reflectance data (points) for four investigated In_2_Te_3_ film thicknesses. Fits and data for the thinnest sample are presented separately in Figure S5. The close agreement between fit and experiment demonstrates the reliability of the extracted amplitude and dephasing time parameters.


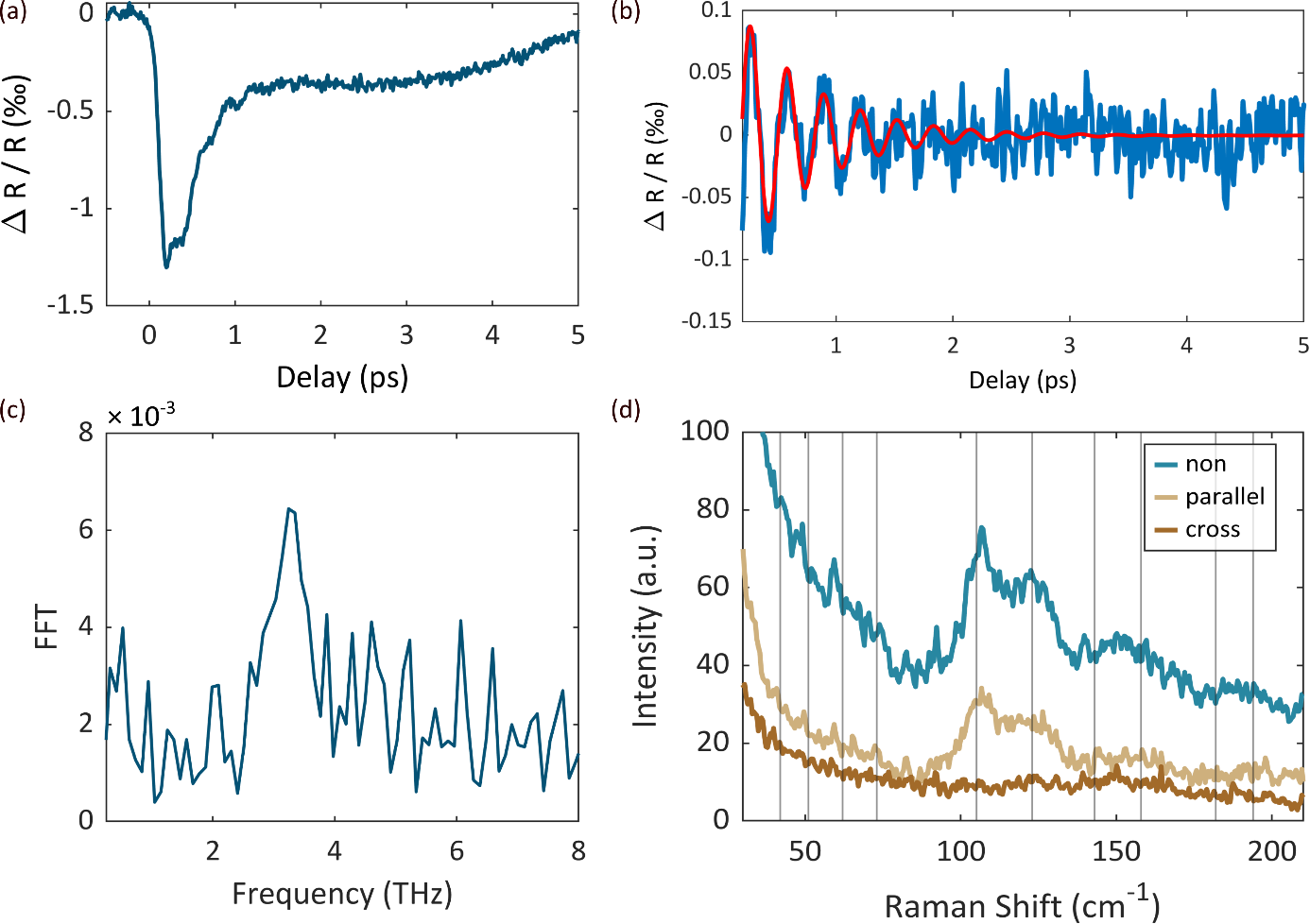


**Figure S8**: Coherent phonon and Raman data for the 2.7 nm In_2_Te_3_ film: (a) Transient reflectance signal; (b) background-subtracted transient reflectance showing coherent phonon oscillations (fit included in red); (c) fast Fourier transform (FFT) of the oscillatory component; and (d) Raman spectra measured in non-polarized, parallel, and cross-polarization configurations. Although the coherent phonon signal is weaker than in thicker films, its presence is clearly observable and further corroborated by polarization-dependent Raman measurements.


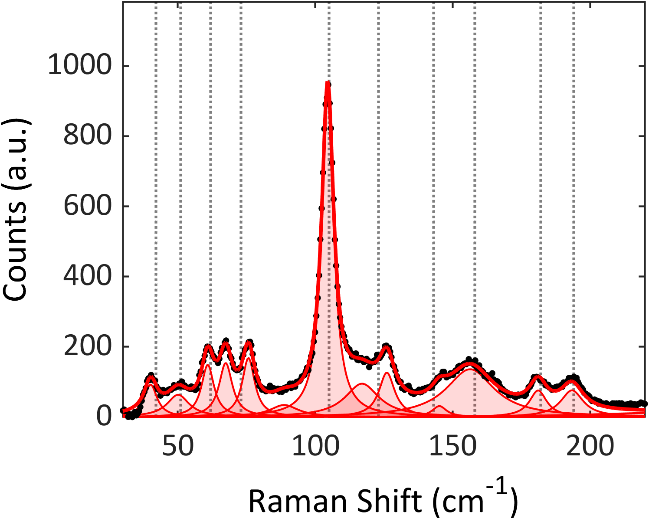


**Figure S9**: Raman data of the 24 nm sample with Lorentzian fits (red) and literature values from [36] (dotted lines).


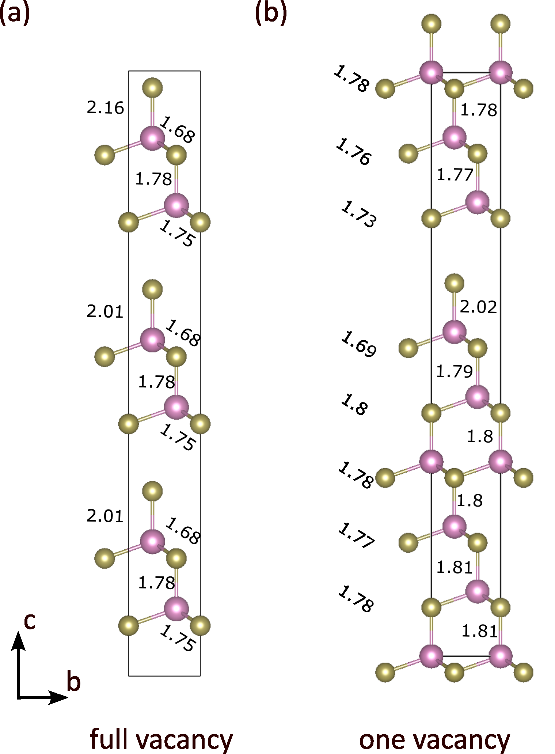


**Figure S10**: Electrons shared (ES) values for two distinct unit cells with different vacancy positions, as homogenous vacancy concentrations cannot be incorporated in these calculations. All ES values fall within a range of 1.6 to 2.2, confirming covalent bonding between the atoms in each unit cell. ES values have been calculated by the DDEC6 method.


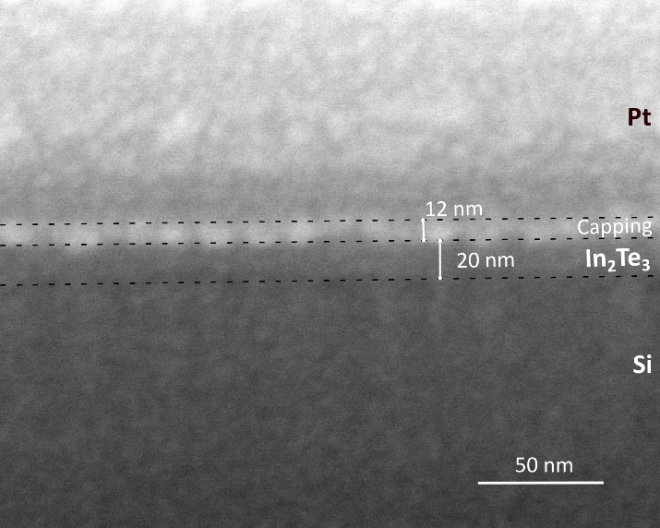


**Figure S11**: For sample preparation, a thin layer of Pt was e-beam deposited on each sample before FIB cross-section milling to protect the surface. The sample stage was tilted to 52°, and we employed a line-by-line cleaning cross-section method using a 30 kV, 40 pA Ga+ FIB source. High-resolution SEM images were then collected at 20 kV, 1.6 nA in immersion mode. However, due to the very low contrast between Si and In_2_Te_3_ in SEM (especially for such thin films) the In_2_Te_3_ layer is not clearly visible in these images.


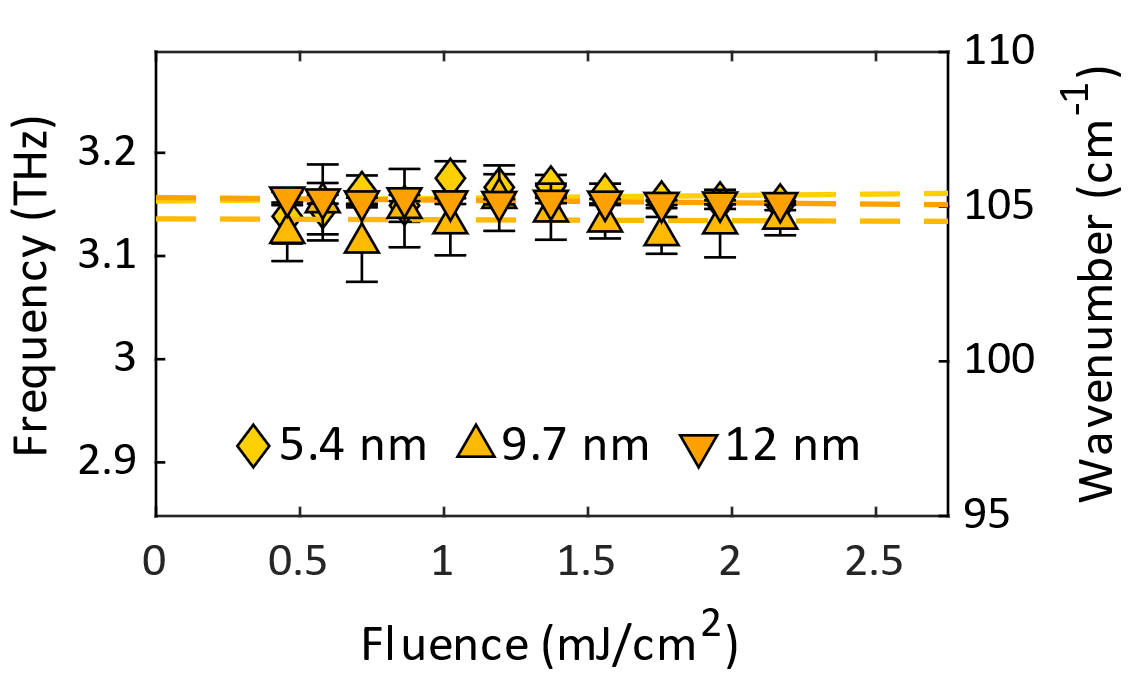


**Figure S12**: Coherent phonon frequencies remain stable across a broad range of incident pump fluences for three different film thicknesses (5 nm to 12 nm). Dashed lines show linear fits, highlighting the absence of phonon softening or hardening with increasing fluence.
